# Macular degeneration affects eye movement behavior during visual search

**DOI:** 10.3389/fpsyg.2013.00579

**Published:** 2013-09-03

**Authors:** Stefan Van der Stigchel, Richard A. I. Bethlehem, Barrie P. Klein, Tos T. J. M. Berendschot, Tanja C. W. Nijboer, Serge O. Dumoulin

**Affiliations:** ^1^Experimental Psychology, Helmholtz Institute, Utrecht UniversityUtrecht, Netherlands; ^2^University Eye Clinic MaastrichtMaastricht, Netherlands; ^3^Rudolf Magnus Institute of Neuroscience and Centre of Excellence for Rehabilitation Medicine, University Medical Centre Utrecht and Rehabilitation Centre De HoogstraatUtrecht, Netherlands

**Keywords:** visual search, macular degeneration, eye movements, preferred retinal location, saccades

## Abstract

Patients with a scotoma in their central vision (e.g., due to macular degeneration, MD) commonly adopt a strategy to direct the eyes such that the image falls onto a peripheral location on the retina. This location is referred to as the preferred retinal locus (PRL). Although previous research has investigated the characteristics of this PRL, it is unclear whether eye movement metrics are modulated by peripheral viewing with a PRL as measured during a visual search paradigm. To this end, we tested four MD patients in a visual search paradigm and contrasted their performance with a healthy control group and a healthy control group performing the same experiment with a simulated scotoma. The experiment contained two conditions. In the first condition the target was an unfilled circle hidden among c-shaped distractors (serial condition) and in the second condition the target was a filled circle (pop-out condition). Saccadic search latencies for the MD group were significantly longer in both conditions compared to both control groups. Results of a subsequent experiment indicated that this difference between the MD and the control groups could not be explained by a difference in target selection sensitivity. Furthermore, search behavior of MD patients was associated with saccades with smaller amplitudes toward the scotoma, an increased intersaccadic interval and an increased number of eye movements necessary to locate the target. Some of these characteristics, such as the increased intersaccadic interval, were also observed in the simulation group, which indicate that these characteristics are related to the peripheral viewing itself. We suggest that the combination of the central scotoma and peripheral viewing can explain the altered search behavior and no behavioral evidence was found for a possible reorganization of the visual system associated with the use of a PRL. Thus the switch from a fovea-based to a PRL-based reference frame impairs search efficiency.

## Introduction

Macular vision is important for tasks that involve high spatial acuity, such as reading and recognizing faces. When central vision is damaged, e.g., due to macular degeneration (MD), people will miss this location of high visual acuity and therefore experience problems with these high-acuity tasks. Both juvenile and age-related MD exist. The juvenile form of MD occurs in 1 out of 10,000 people (Bither and Berns, 1985), whereas age-related MD is the main cause of diminished visual acuity in the elderly (Leibowitz et al., 1980). In its advanced stage, MD creates a central visual field scotoma. Therefore, MD patients typically adopt a strategy to direct the eyes such that the image falls onto a peripheral location on the retina to compensate for their impairment: a location on the retina which is not part of the scotoma and which functions as a “pseudo-fovea” (Timberlake et al., 1986, 1987; Whittaker et al., 1988; Fletcher et al., 1999). Currently, there is a debate whether this strategy coincides with a reorganization of the visual system (Baker et al., 2005, 2008; Masuda et al., 2008).

Similarly, the oculomotor system needs to adapt to this PRL as well. Eye movements bring regions of interest toward the fovea. In MD a PRL-based reference frame replaces the fovea-centered reference frame. Therefore, eye movements in MD patients might be expected to be less effective than in normally sighted people. Although previous studies have indicated that a shift of oculomotor reference to a non-foveal location is indeed possible, eye movements using this new oculomotor reference are generally associated with prolonged initiation latencies and decreased accuracy (White and Bedell, 1990; Whittaker et al., 1991). Furthermore, the fixation ability is known to be impaired when a new oculomotor reference frame is adopted, although this ability can be trained resulting in improved reading abilities (Tarita-Nistor et al., 2009).

Whereas previous studies have examined the oculomotor behavior of the PRL in reading tasks in detail (McMahon et al., 1991; Fletcher et al., 1999; Lingnau et al., 2008; Chung, 2011; Nguyen et al., 2011), very little is known about the characteristics of oculomotor behavior in other tasks such as visual search. Reading is a specific oculomotor task, which includes numerous constraints such as the horizontal lay-out of a text and the small saccades associated with foveating words. We suggest that visual search can be considered a more naturalistic viewing task, which requires more exploratory oculomotor behavior. Knowledge about the oculomotor behavior of the PRL, such as saccade amplitude and search times, in visual search tasks might therefore unravel the characteristics of the eye movement pattern in daily life situations that do not require reading. Although previous studies have revealed that visual search is impaired in persons with a visual impairment, including age-related MD (Kuyk et al., 2005; MacKeben and Fletcher, 2011), the underlying oculomotor behavior specific to the PRL remains unclear.

The aim of the current study was to examine the oculomotor search behavior of MD patients with a new oculomotor reference frame. To this end, four patients with a stable PRL due to juvenile MD participated in a search task while their eye movements were recorded. The performance of this group during visual search was quantified in terms of search time, saccade amplitudes and number of saccades needed to bring the target to the PRL. This performance was compared to the search behavior of a group of normally sighted participants and a group of normally sighted participants who performed the task with a simulated central scotoma (and therefore viewed the search array with their peripheral vision, Henderson et al., 1997). This way, we were able to compare the PRL-based reference frame with a fovea-based reference frame either using the fovea or a peripheral location. Previous studies have revealed that inducing a simulated central scotoma in normally sighted participant induces impairments in visual search tasks, such as increase in search time and fixation duration (Bertero, 1988; Cornelissen et al., 2005; McIlreavy et al., 2012) and a decrease of search facilitation for repeated displays (Geringswald et al., 2012). To compare the search behavior in different types of visual search, the experiment contained two conditions: in the first condition the target was an unfilled circle hidden among c-shaped distractors of equal size (serial condition) and in the second condition the target was a filled circle (pop-out condition). These two search conditions are associated with different search behavior in that the serial condition will result in slow and serial search, whereas the pop-out condition will result in rapid, parallel search (Van der Stigchel et al., 2009). This pop-out condition will unravel whether fast parallel visual search is still possible when a new oculomotor reference frame is adopted.

## Methods

### Participants

Four patients with central vision loss participated in the experiment: Cases 1, 2, 3, and 4 (Table 1). Ten healthy controls (age 29.9 ± 10.0, 4 males) participated in the visual search task. Five healthy controls (age 25.2 ± 1.3, 3 males) were recruited to perform a visual search task with an online scotoma simulation. The participants gave informed consent and this study was approved by the ethical committee of the University Medical Center Utrecht.

**Table 1:** **Patient descriptives**.

**Patient**	**Gender**	**Diagnosis**	**Age (year)**	**Age diagnosis (year)**	**Dominant eye**	**PRL Eccentricity**
Case 1	Female	Stargardt	29	12	Left	15.5°
Case 2	Male	Stargardt	23	9	Left	2.3°
Case 3	Male	Stargardt	47	40	Right	10.9°
Case 4	Female	Stargardt	21	8	Left	
Case 5	Female	Stargardt	31	15	Right	
Case 6	Female	Stargardt	29	9	Left	
Case 7	Female	Stargardt	19	17	Right	

### Equipment

We used a Iiyama CRT display monitor (subtending 36.83 × 27.62°; refresh rate 60 Hz), driven by a Dell Precision PWS390 computer for all experiments. The contrast was kept as high as possible to enhance visibility. Eye movements of the dominant eye were registered with a sampling frequency of 1000 Hz using the Eyelink 1000 infrared eye-tracker (Desktop Mount; SR Research Ltd.). Thresholds for detecting the onset of a saccadic movement were acceleration of 8000°/s^2^ and a velocity of 30°/s.

### Stimulus and procedure

Prior to the experiment, the participants' dominant eye was determined using a visual alignment task (Porac and Coren, 1976). Participants sat in front of the computer monitor with their eyes at a distance of 57 cm from the screen, while their chin rested in a chin rest. Each participant was given a verbal explanation of the tasks they were about to perform. The experiment started directly after calibration of the eye-tracker. Using a nine-point grid calibration participants were asked to fixate on the calibration dots. In the case of the MD-group, participants were explicitly instructed to try and see the dot as clear as possible. During the calibration procedure the calibration was validated by presenting the nine calibration points again. The calibration was only successful with a small error between the two presentations for each calibration point (<1°). This procedure ensures that patients were using the same retinal location during calibration for a specific calibration point. Because patients were viewing a calibration point consistently with the same retinal location, we assume that patients were fixating with their PRL.

Before each experimental procedure started, participants were given eight practice trials. Each trial started with a drift check. Only trials with an oculomotor drift smaller than 2° were accepted. Directly after the drift check, the trial started^1^. The targets and distractors in all tasks were black and projected on a white background (80.9 cd/m^2^). The Michelson contrast was 98%.

### Visual field and SLO measurement

**Figure 1:**
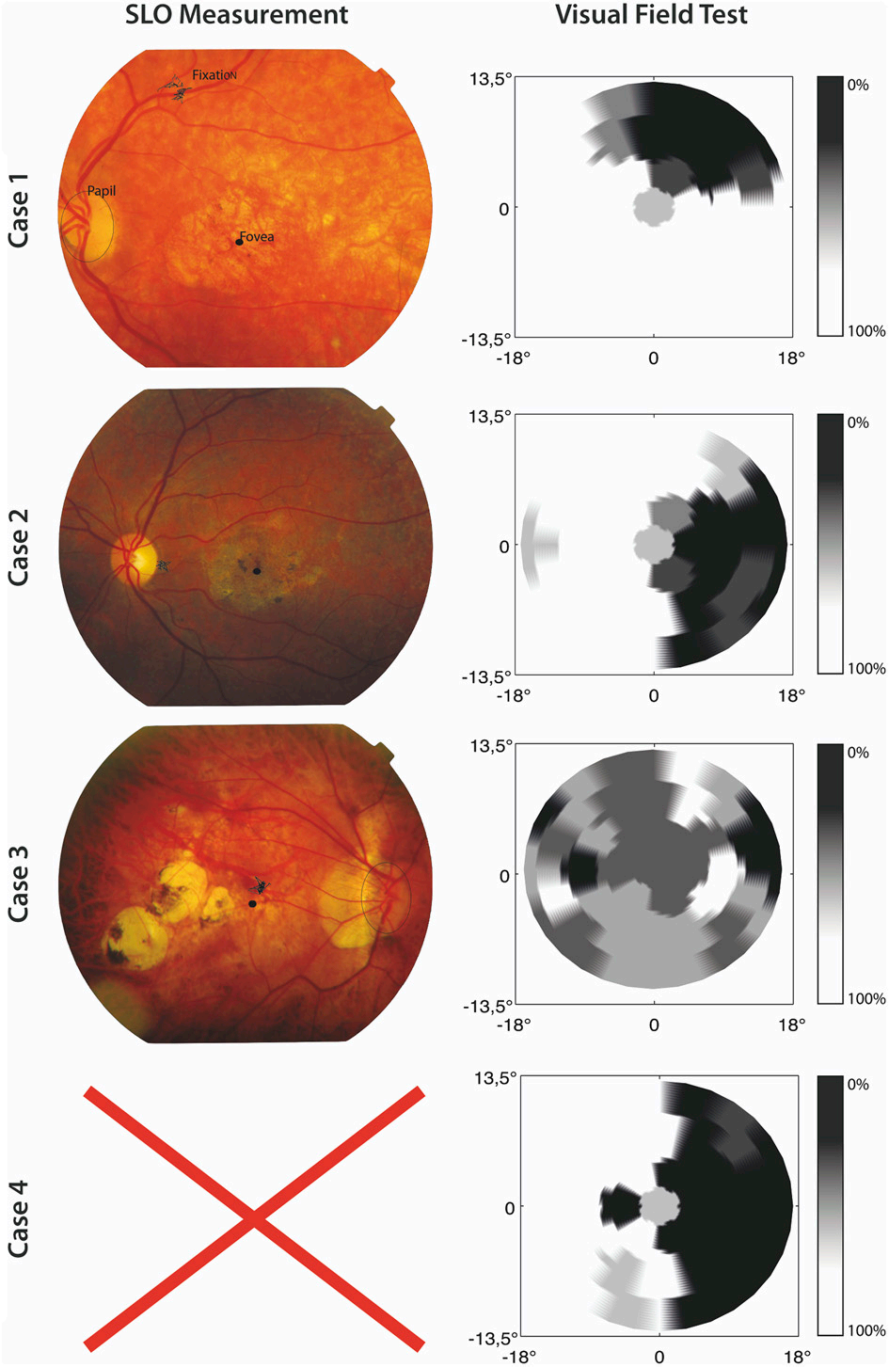
**SLO Measurement and results of the visual field test for the included MD patients**. The left panel shows SLO pictures where fixation, fovea, and papilla are marked. No SLO measurement was possible for Case 4. For the results of the visual field test the gray scale indicates the percentage correctly identified targets.

A visual field test was used to check if participants in the MD group indeed suffered from a central scotoma. In this visual field test, only one target at a time was shown and a fixation cross was used which remained on screen during presentation of the target. The target was a 1.5° circle and could appear at 33 possible stimulus locations. The 33 locations were organized in five rows; three rows consisted of seven locations and two rows of six locations, corresponding to the target locations used in the visual search paradigm discussed later. Participants were instructed to remain fixated on the fixation cross and report, using the “z” and “/” keys, whether they had seen a target or not. After their response a confirmation of their choice was presented on screen. Target present trials were mixed with “catch” trials in which no target was presented. All target locations were presented four times along with 16 catch trials, making for 148 trials in total. The visual field test confirmed that all MD patients suffered a partial scotoma; all patients reported to have not seen a target for certain locations tested in the visual field test. No patient reported false positives. In one case (Case 3) the visual field test was not useful in clearly determining the scotoma, because the loss of vision was not limited to a specific quadrant (see Figure 1).

Furthermore, in order to gain information about fixation stability and absolute locus of the PRL, the MD patients were invited for a Scanning Laser Ophthalmoscope (SLO) measurement at the University Eye Clinic Maastricht. We used a home build SLO with the ability to modulate the laser intensity (Ossewaarde-Van Norel et al., 2002) which enabled us to project a fixation cross in the raster pattern. To determine the absolute location of the PRL at the retina and its stability, 60 SLO images per patient were acquired with the use of a frame grabber, having a 1 sec interval in between. The SLO measurement showed clear peripheral fixation. One case (Case 4) was unable to participate in an SLO measurement due to technical difficulties. For the three remaining cases, fixation stability was, on average, within 1.2° standard deviation of the fixation point. Figure 1 shows the results from the visual field test and SLO measurement.

### Visual search task

**Figure 2:**
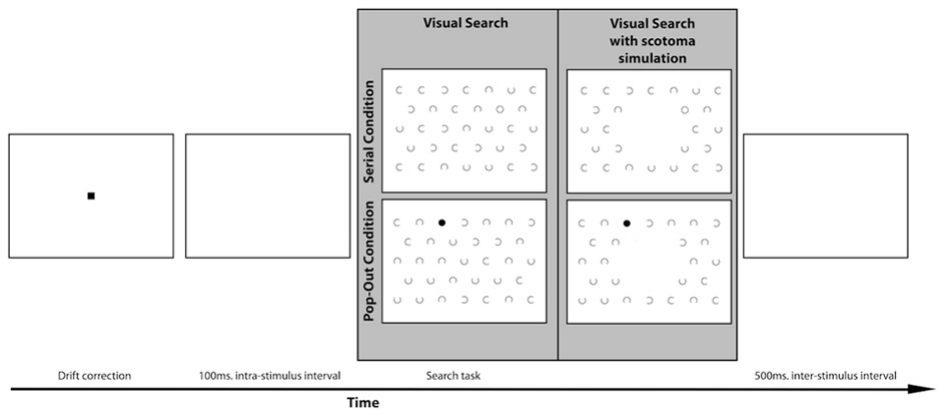
**Lay-out of the visual search paradigm with an example of a trial in both the serial and the pop-out condition**. The gray panel shows an example of a search display for the MD and the control group on the left. Participants in the simulation group performed the visual search task with a simulated central scotoma, as indicated on the right of the gray panel.

The stimulus for the visual search task consisted of a visual search field with 32 c-shaped distractors and one target. There were two conditions, the target being either a circle or a dot, see Figure 2. Both the location of the target and the orientation of the opening of the C's varied for each trial. The openings of the C's could be oriented toward the top (0°), right (90°), bottom (180°), or left (270°). The objects were organized in the same manner as the visual field paradigm. The target could appear at all locations except the center location and the six locations around the center. The target could therefore appear at 26 locations. The objects were 1.5° wide, and the C's and the target circles' rim size was 0.1°. The target dots had the same size as the C's and the target circles, but were filled. The size of the gaps of the C's was 0.93°. The objects were placed at a center-to-center distance of 5°. This was the case within the rows as well as between the rows. The search field was presented 100 ms after the drift correction. During the 100 ms interval a white screen was presented. Participants were instructed to find the target as soon as possible. They were explicitly instructed to fixate on the target as soon as they found it. When they had found the target, participants pressed the space bar to continue to the next trial. Every target-type was used two times on every target location. In total the visual search task contained 110 trials.

A separate group of healthy controls participated in a scotoma simulation version of the visual search task. In this version of the visual search task the center of the visual field was occluded online with a 10° disc that had the same luminance and color as the background to simulate a central scotoma. Targets could also appear inside the area of simulated scotoma with equal probability as the other areas in the search array. After calibration of the eye-tracker this dot “followed” the participant's eye movement in real-time continuously occluding their center of vision, see Figure 2.

### Data analysis

Saccades were detected off-line and only saccades larger than 0.5° were accepted. In the visual search analysis, saccades larger than 40° were excluded from the analysis, because this is larger than the search field. Only trials were accepted in which a saccade was made onto the target. A saccade on the target was defined as a saccade that ended on a location within a radius of 1.97° of the center of the target. Trials on which a saccade was made within 80 ms after stimulus presentation were also excluded from further analysis. Trials for which the total search latencies exceeded two standard deviations from the subject mean were also excluded. This led to an average exclusion of 4.0% of the trials in the control group, 6.7% in the MD group and 14.4% in the simulation group. For the remaining trials search latency was analyzed as the primary measure. Search latency is defined as the time between stimulus onset and the moment the eye landed on the target. In addition to search latency, saccade amplitudes, number of saccades, and inter-saccade-interval of all saccades until the target was found were analyzed.

One-Way repeated measures ANOVA was used to determine main group (Control × Simulation × MD) effects for both conditions separately. Where Levene's test for homogeneity of variances indicated that homoscedascity was violated, Brown–Forsythe robust test for equality of means was used instead. *Post-hoc t*-tests with Bonferroni correction for multiple comparisons were used to make *post-hoc* group comparisons.

## Results

### Search latency

**Figure 3:**
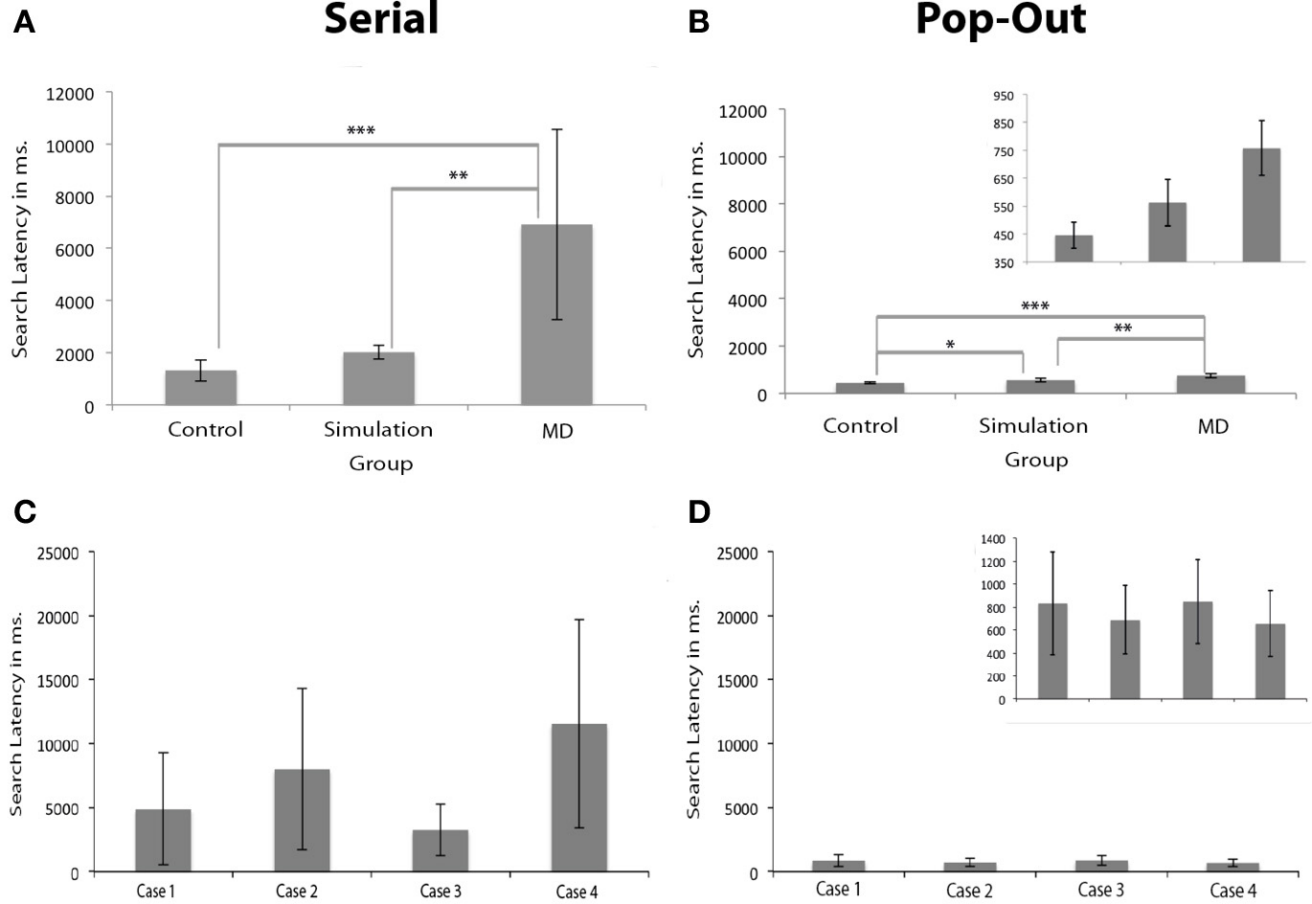
**Search latencies in visual search. (A,B)** Show the search latencies for the *serial* and *pop-out* condition. Error bars represent the groups standard deviation. **(C,D)** Show the individual search latencies for Cases 1 to 4 (here, error bars represent individual standard deviations). The figures for the pop-out condition are presented twice: once with the same range as the serial condition and once with an adapted range for visibility of the differences between the groups and the individual subjects. The MD group had longer search latencies than the simulation and the control group, irrespective of whether the target was presented in the scotoma. Significant differences are marked (± meaning a trend level *p*-value, ^*^p < 0.05, ^**^p < 0.01, and ^***^p < 0.001).

Results show that search latency, defined as the time required to find a target, was longer in the MD group. Brown–Forsythe robust test for equality of means indicated a trend level effect of group on search latency in the *serial condition*, *F*_(2, 3.074)_ = 8.733, *p* = 0.054. *Post-hoc* tests revealed that the control group had significantly shorter response latencies in the serial condition compared to the MD group (*p* < 0.001), see Figure 3. Individual subjects are shown in **(C,D)**. The group that performed the scotoma simulation test also indicated faster responses compared to the MD group (*p* < 0.01). There were no differences in search latency in the serial condition between the control group and simulation group. Brown–Forsythe robust test for equality of means also indicated a significant main effect of group on search latency in the *pop-out condition*, *F*_(2, 7.711)_ = 20.365, *p* < 0.01. *Post-hoc* tests revealed that the controls were significantly faster than the MD group (*p* < 0.001) and also faster than people that performed the simulation (*p* < 0.05). Additionally the MD group was significantly slower than the simulation group (*p* < 0.01).

The search latency of the MD group was longer than the control group and simulation group. Based on signal detection theory this may be caused by either a decreased sensitivity or a change in response bias. The latter case may be interpreted as the MD subjects being more “careful” to compensate for their loss in visual acuity. To make sure that these differences in search latency did not originate from a change in response bias a second experiment was performed. In this second visual search paradigm a condition without any target was added to the experimental design and participants were asked to press “z” or “/” on a keyboard to indicate whether the target was present or absent, respectively. Cases 4, 5, 6, and 7 (listed in Table 1) participated in this experiment. Signal detection theory measures were calculated for this second experiment. These indicated that there were no significant differences between the control group (mean *d*' = −0.21; st. dev. = 0.23) and the MD group (mean *d*' = −0.05; st. dev. = 0.15) on choice sensitivity [paired samples *t*-test: *t*_(6)_ = 1.196, *p* = 0.277] nor on bias [*t*_(6)_ = 1.036, *p* = 0.340]. This control experiment indicated that there was no change in response bias, and that the differences in search latency between the control and the MD group can be attributed to decreased sensitivity.

Given that the differences in search latency could not be explained by a selection bias, the oculomotor search behavior was explored to reveal which characteristics caused the increased search latency in the MD group. Here, we examined saccade amplitude, number of saccades needed to foveate the target and the intersaccadic interval.

### Saccade amplitude

**Figure 4:**
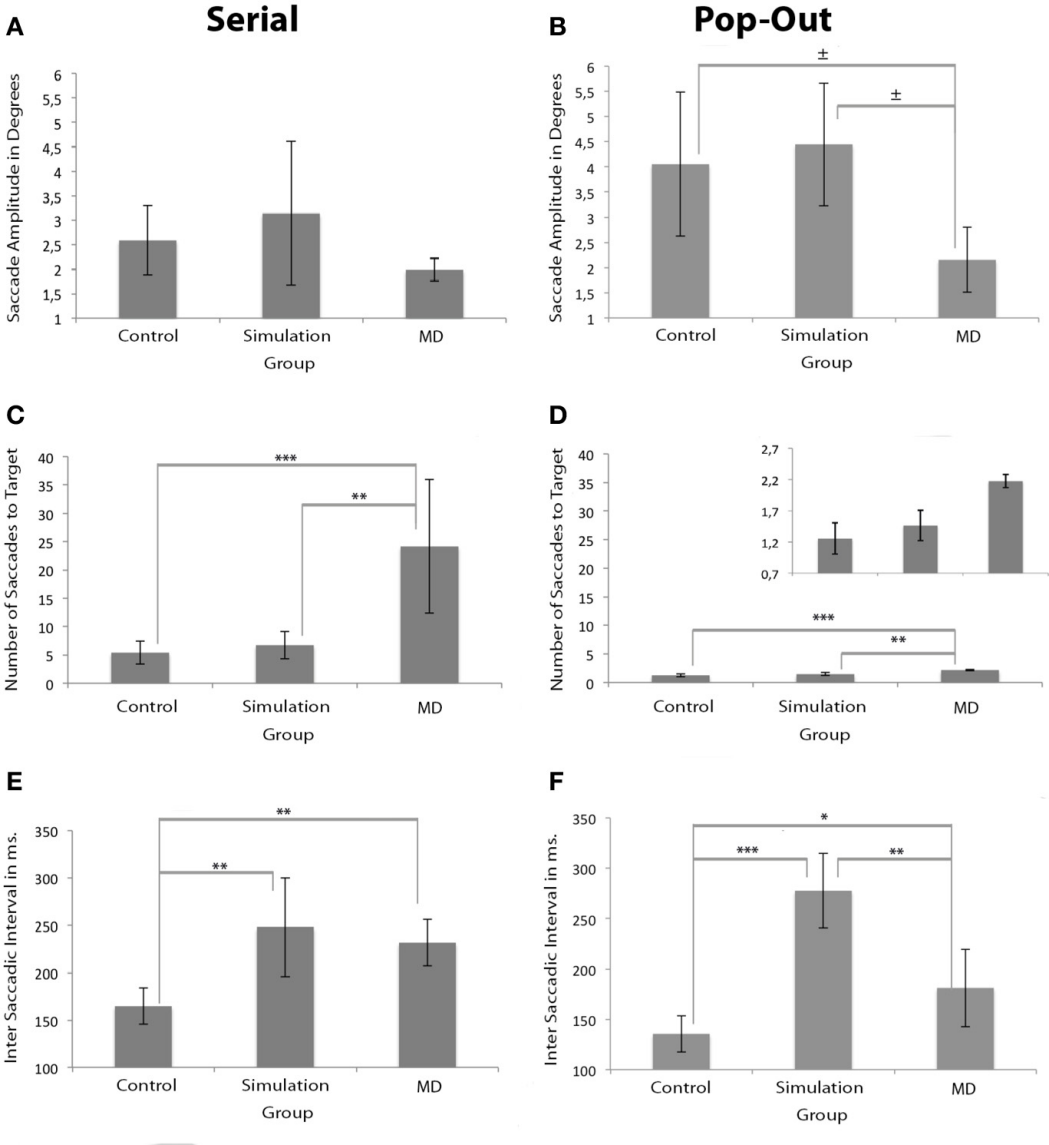
**Measurements of oculomotor characteristics**. Left and right panels show the results for the serial and pop-out conditions. **(A,B)** Show saccade amplitudes. **(C,D)** Show the number of saccades before the eye landed on the target for both conditions. The figure of the number of saccades for the pop-out condition is presented twice: once with the same range as the serial condition and once with an adapted range for visibility of the differences between the groups. **(E,F)** Show the inter-saccade-interval in milliseconds for both conditions. Significant differences are indicated (^*^p < 0.05, ^**^p < 0.01, and ^***^p < 0.001; ^±^ 0.05 > *p* <0.07).

The amplitude of saccades for the MD group was similar to both control groups in the *serial* condition, but not in the *pop-out* condition. Brown–Forsythe indicated no main group effect on average saccade amplitude in the *serial condition*. There was a main effect of group on average saccade amplitude in the *pop-out condition* as apparent from One-Way repeated measures ANOVA, *F*_(2, 17)_ = 4.210, *p* < 0.05, see Figures 4A,B. *Post-hoc* tests indicated a trend that the control group made larger saccadic movement than the MD group in the *pop-out condition* (*p* = 0.068). There was no significant difference between the control and the simulation group in the *pop-out condition*. There was a trend level difference between the MD group and the simulation group, revealing that the MD group made, on average, smaller saccadic movements (*p* = 0.056).

### Number of saccades to target

MD patients needed a higher number of saccades to find the target. Brown–Forsythe tests indicated a significant effect of group on number of saccades to a target *F*_(2, 3.354)_ = 9.243, *p* < 0.05 in the *serial condition*, see Figure 4C. *Post-Hoc* tests indicated that the control and the simulation group needed significantly fewer saccades than the MD group (*p* < 0.001 and *p* < 0.01 respectively). One-Way repeated measures ANOVA also revealed a main effect of group on number of saccades to target in the *pop-out condition*, *F*_(2, 17)_ = 23.713, *p* < 0.001, see Figure 4D. *Post-hoc* tests indicated that the MD group needed more saccades to find a target compared to the control group (*p* < 0.001) and the simulation group (*p* < 0.01).

### Inter saccadic interval

Compared to the control group, the inter saccadic interval was significantly longer in the simulation group in both conditions. In the *serial* condition the MD group also showed longer saccadic intervals. Brown–Forsythe tests indicated a significant main effect of group on inter-saccadic-interval *F*_(2, 4.795)_ = 9.373, *p* < 0.05 in the *serial condition*. *Post-Hoc* tests reveal that in the *serial condition* the simulation group had significantly longer inter-saccadic-intervals compared to the control group (*p* < 0.01), see Figure 4E. Compared to the control group, the MD group also had a significantly longer inter-saccadic-interval (*p* < 0.01). In the *pop-out condition* One-Way repeated measures ANOVA indicated a significant effect, *F*_(2, 17)_ = 37.470, *p* < 0.001, Figure 4F. *Post-Hoc* tests revealed that in this condition the simulation group used longer intervals than the control (*p* < 0.001) and the MD group (*p* < 0.01).

### Saccade direction

**Figure 5:**
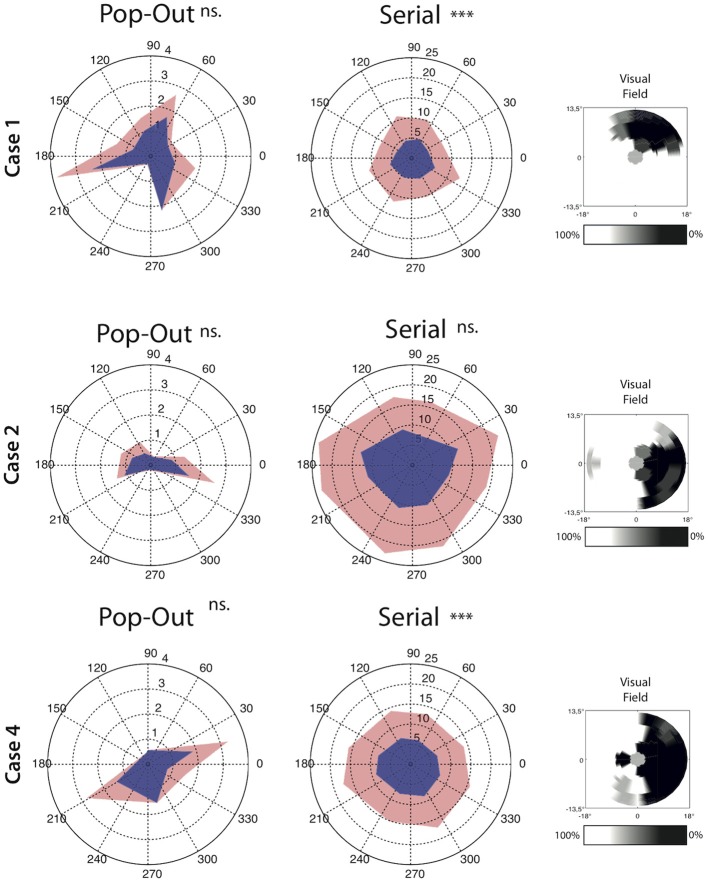
**Average angle by direction characteristics for all saccades for each condition separately**. Saccade amplitude is depicted as the radius whereas the angle represent the direction of the saccades. Upper confidence interval is plotted in light-red on top of the average saccade amplitude. Note that the left and middle panel have a different range. The right panel for each case depict the interpolated scotoma based on measurements from the visual field test. Cases 1 and 4 show lower saccade amplitude toward the scotoma than away from the scotoma in the serial search condition. Significant differences are indicated (^***^p < 0.001, ns. = non-significant).

In addition to the measures described above we also set out to analyse if the saccade amplitude was different for saccades directed toward the scotomous area compared to saccades moving away from the scotoma. In three patients we could clearly and reliably define one or two quadrants of the visual field as scotomous (see Figure 5). Within each participant and each condition we compared saccades toward and away from the scotomous area. For saccade amplitude, both Case 1 and Case 4 had larger saccade amplitudes away from the scotoma compared to saccades towards the saccade amplitude in the *serial condition* [for Case 4; *t*_(1376)_ = −3.34, *p* < 0.001, for Case 1; *t*_(580)_ = −4.66, *p* < 0.001]. There were no significant differences in the *pop-out condition* for any of the patients.

## Discussion

The aim of the present study was to examine the oculomotor behavior of MD patients who developed a stable PRL to explore the visual world. To this end, four MD patients participated in a visual search experiment. Their performance was compared to a control group and a control “simulation” group in which a central scotoma was simulated. Search performance was impaired for MD patients: the search latency, the time necessary to locate and foveate the target, was longer for the MD group than for the control group. This was observed for both the serial and the pop-out search conditions. Although participants in the simulation group were somewhat slower than the control group, this group outperformed the MD group in both the serial and the pop-out search conditions. Control experiments indicated that the differences in search latency between the control and the MD group were not caused by a difference in selection bias.

To investigate the source of the slowing in visual search in MD patients, oculomotor behavior in the search task was divided in three separate measures: saccade amplitude, the number of saccades needed to foveate the target and the intersaccadic interval (i.e., the time in between fixations). With respect to saccade amplitude, no differences were observed in the serial condition between the different groups. However, when taking saccade direction into account relative to the scotoma, saccades toward the scotoma had a smaller amplitude than saccades away from the scotoma. This effect was observed in two of the three cases in which this analysis was possible. Thus saccade amplitude seems affected in a direction-specific manner, an effect lost when pooling over all directions. In the pop-out condition, MD search behavior was also associated with smaller saccadic amplitudes. No effect of saccade direction was found, but this might have been a power issue as the total number of saccades in the pop-out search condition was much lower than in the serial search condition. In short, MD search behavior is associated with smaller saccade amplitudes.

When examining the number of saccades, the results indicated that the MD group made more saccades, both in the pop-out and the serial condition. Finally, the intersaccadic interval was longest in the MD and the simulation group compared to the control group. These results were most reliable in the serial condition, as the pop-out search condition only indicated a main effect at a trend level. In short, we attribute the increased search latencies in MD to decreased saccadic amplitudes, increased number of saccades and increased saccadic intervals.

The simulation group cannot be considered the ideal comparison to the behavior observed in the MD group. Firstly, the scotoma is always visible to control subjects which might influence oculomotor programming. Secondly, MD is not only associated with a central scotoma (absolute scotoma), but will also affect regions beyond the scotoma to various degrees (relative scotoma). Thirdly, the location of the PRL might not be the location of highest acuity on the residual retina (Shima et al., 2010). This was also reflected in the oculomotor behavior of the MD group: although they have developed a new oculomotor reference frame, search latency was longer than the simulation group. If the sole difference between the simulation and the MD group was the central scotoma, one might expect the MD group to outperform the simulation group, because the MD group has more experience with viewing the visual world with a central scotoma than the simulation group. As this was not observed, we suggest that part of the overall slowing in search performance is due to more diffuse impairments of retinal functions. Besides the diffuse loss of retinal function, the search time is also increased by the presence of a central—absolute—scotoma: the simulation group was slower than the control group, most prominently in the pop-out condition.

In line with the previous observation that the absolute scotoma affects search performance, some of the MD participants made saccades with shorter amplitudes toward the scotoma than away from the scotoma. This can be explained by the fact that visual information projected to the scotomous region is not being transferred to the oculomotor system. The “selection for-action” view (Schneider and Deubel, 1995, 2002) argues that the function of spatial attention is to provide a spatial code to the eye movement system to program the subsequent eye movement. As spatial attention cannot be allocated to the scotomous region, a bias might therefore develop to not make eye movement toward the scotomous region, resulting in saccades with shorter amplitudes which still land outside the region of the visual field previously subserved by the scotoma. The shorter amplitudes in the MD group might also be related to the increased number of eye movements necessary to find the target; with shorter eye movements, it takes more saccades to make a complete search of the visual field.

The idea that spatial attention cannot be allocated to the scotomous region is reminiscent of a study in which normal-sighted participants performed a visual search task while vision was restricted to a gaze-contingent viewing window (Lingnau et al., 2010). This study varied the location of the viewing window and found no specific part of the visual field or gaze direction for which performance on the visual search task was most beneficial. Interestingly, however, performance on the visual search task was directly related to the location of the viewing window and the direction of visual attention: there was an impaired performance when visual attention and gaze had to be moved in opposite directions. In line with our results, this appears to indicate that there is a direct interplay between the direction of visual attention and the location of the scotoma.

As our eye tracker was calibrated with respect to the macula in the simulation group and they did not have a stable PRL, a similar analysis could not be performed for the simulation group and it was impossible to disentangle between eye movements toward and away from the scotoma. However, as no overall decrease in saccade amplitude was observed in the simulation group compared to the control group, it is possible that this decrease in saccade amplitude is restricted to the MD group. We speculate that it could be beneficial to make saccades with a higher amplitude toward the scotoma, because this will bring more visual information from the scotoma to the region with detailed vision. Future studies focusing on training oculomotor behavior in MD patients might include this factor to investigate whether this is truly beneficial or whether the observed shorter saccades is the most optimal solution.

The intersaccadic interval observed in the MD group was similar to the interval observed in the simulation group, whereas both groups had longer intervals compared to the control group. This result therefore seems to be specific to peripheral processing of the target. The periphery may need more time to process the stimulus information than the fovea. Interestingly, the overall decrease in visual acuity in the MD group is expected to result in an even longer interval as it is expected to take longer to process visual information when visual acuity in the periphery is low than when visual acuity is high, like in the simulation group. The comparable interval between the MD and the simulation group might therefore be the result of a large amount of experience using the PRL for MD patients.

Our results reveal no evidence for the claim that the use of a PRL coincides with a reorganization of the visual system (Baker et al., 2005, 2008; Masuda et al., 2008). Presumably, if the adult visual system has compensatory mechanisms to cope with adventitious loss, its ultimate goal would be to have beneficial behavioral consequences. Reorganization and in particular access of the PRL to fovea-based cortical processing units might have resulted in an improved search performance of the MD patients with respect to the simulation group, who have no experience with a central scotoma. This was not observed. This conclusion should be regarded with caution, however, as the simulation group had no peripheral retinal impairments, in contrast to the MD patients. Thus any possible benefit from a reorganization of the visual system was not able to overcome the impaired performance associated with the loss of retinal function.

To conclude, search behavior is impaired in MD patients and is associated with saccades with decreased amplitudes toward the scotoma, an increased intersaccadic interval and an increased number of eye movements necessary to locate the target. This suggests that the switch from a fovea-based to a PRL-based reference frame comes at the cost of search efficiency.

### Conflict of interest statement

The authors declare that the research was conducted in the absence of any commercial or financial relationships that could be construed as a potential conflict of interest.

## Footnotes

^1^Although the drift check failed more frequently in the MD group than the other groups (17 vs. 2%, respectively), search latency in the MD group did not differ between trials in which the drift check failed and trials in which the drift check was immediately successful (*p*'s > 0.25).
